# The Potential Role of Gut Microbiota in Alzheimer’s Disease: From Diagnosis to Treatment

**DOI:** 10.3390/nu14030668

**Published:** 2022-02-05

**Authors:** Angelica Varesi, Elisa Pierella, Marcello Romeo, Gaia Bavestrello Piccini, Claudia Alfano, Geir Bjørklund, Abigail Oppong, Giovanni Ricevuti, Ciro Esposito, Salvatore Chirumbolo, Alessia Pascale

**Affiliations:** 1 Department of Biology and Biotechnology, University of Pavia, 27100 Pavia, Italy; drmarcelloromeo@gmail.com; 2 Almo Collegio Borromeo, 27100 Pavia, Italy; 3 School of Medicine, Faculty of Clinical and Biomedical Sciences, University of Central Lancashire, Preston PR1 2HE, UK; epierella1@uclan.ac.uk (E.P.); aoppong1@uclan.ac.uk (A.O.); 4 Emergency Medicine, Université Libre de Bruxelles, 1050 Brussels, Belgium; gaia.bavestrellopic01@universitadipavia.it; 5 Department of Emergency Medicine and Surgery, IRCCS Fondazione Policlinico San Matteo, 27100 Pavia, Italy; claudia.alfano86@gmail.com; 6 Council for Nutritional and Environmental Medicine (CONEM), 8610 Mo i Rana, Norway; geir@vitalpress.no; 7 Department of Drug Sciences, University of Pavia, 27100 Pavia, Italy; 8 Unit of Nephrology and Dialysis, ICS Maugeri, University of Pavia, 27100 Pavia, Italy; ciro.esposito@unipv.it; 9 Department of Neurosciences, Biomedicine and Movement Sciences, University of Verona, 37121 Verona, Italy; salvatore.chirumbolo@univr.it; 10 Section of Pharmacology, Department of Drug Sciences, University of Pavia, 27100 Pavia, Italy; alessia.pascale@unipv.it

**Keywords:** Alzheimer’s disease, gut microbiota, dysbiosis, gut–brain axis, biomarker, prebiotics, probiotics, diet, fecal microbiota transplantation

## Abstract

Gut microbiota is emerging as a key regulator of many disease conditions and its dysregulation is implicated in the pathogenesis of several gastrointestinal and extraintestinal disorders. More recently, gut microbiome alterations have been linked to neurodegeneration through the increasingly defined gut microbiota brain axis, opening the possibility for new microbiota-based therapeutic options. Although several studies have been conducted to unravel the possible relationship between Alzheimer’s Disease (AD) pathogenesis and progression, the diagnostic and therapeutic potential of approaches aiming at restoring gut microbiota eubiosis remain to be fully addressed. In this narrative review, we briefly summarize the role of gut microbiota homeostasis in brain health and disease, and we present evidence for its dysregulation in AD patients. Based on these observations, we then discuss how dysbiosis might be exploited as a new diagnostic tool in early and advanced disease stages, and we examine the potential of prebiotics, probiotics, fecal microbiota transplantation, and diets as complementary therapeutic interventions on disease pathogenesis and progression, thus offering new insights into the diagnosis and treatment of this devastating and progressive disease.

## 1. Introduction

Alzheimer’s disease (AD), which affects approximately 50,000,000 people worldwide, is the most frequent cause of dementia, constituting a real global health problem [1]. The disease is characterized by the progressive deposition of beta amyloid (Aβ) plaques and tangles of hyperphosphorylated tau neurofibrils, leading to neuroinflammation and progressive cognitive decline [2]. Synaptic dysfunction and neuronal death are at least in part due to the excessive or non-resolving activation of the immune response and any infections or traumatic events affecting the brain (traumatic brain injury) can interfere with central immune homeostasis and accelerate the progression of the disease [3]. Although several hypotheses have been formulated about the causes of AD pathogenesis and progression, both the onset and the evolution of the disease remain not entirely clear. Therefore, although different therapeutic options have been proposed, many have failed in clinical trials and have not been found to produce significant benefits [4,5,6]. It is widely thought that an early diagnosis could be essential to act at the earliest disease stages, but effective and reproducible biomarkers are still far from clinical application [7,8]. 

In recent years, the gut microbiota brain axis (GMBA) has been at the center of biomedical research and it has been suggested as a potential therapeutic target for disorders affecting the central nervous system, including AD [9,10,11].

The term “gut microbiota” refers to the commensal microbial community that colonizes the gastrointestinal tract and is constituted by bacteria, fungi, archaea, viruses, and protozoans living in symbiotic relationship with our intestine [9,12,13,14]. Thanks to their active role in regulating host’s homeostasis and disease, they are becoming more and more important in the pathogenetic mechanisms of neurodegenerative disorders, such as AD [15,16,17,18]. Indeed, even though for a long time it was believed that the brain was a totally isolated organ, recent evidence shows that the gut microbiota is at the center of a bidirectional communication between intestine and brain, the so-called microbiota gut–brain axis [15,19,20,21]. This interplay involves the central nervous system (CNS), the autonomic nervous system, the enteric nervous system (ENS), and the hypothalamus-pituitary-adrenal axis (HPA), and it has been reported to be implicated in a number of physiological and pathological processes such as satiety, food intake, glucose and fat metabolism, insulin sensitivity, and stress [22]. Although the mechanisms underlying this interaction are not fully understood, targeting the microbiota might represent a new diagnostic and therapeutic strategy in AD and in other neurodegenerative diseases [23]. However, despite several published papers having reviewed possible microbiome-based therapies, to our knowledge a comprehensive view of gut microbiota-based diagnostic and therapeutic approaches is still lacking. Here, based on the main studies addressing gut microbiota dysregulation in AD, we discuss how the microbiota-derived biomarkers might be exploited for early disease detection, and we review the potentiality of probiotics, prebiotics, diet, and fecal microbiota transplantation as complementary therapeutic options for this devastating and progressive disease. 

## 2. Main

### 2.1. The Gut–Brain Axis: An Overview

The gut–brain axis (GBA) consists of a signaling pathway between the gastrointestinal (GI) tract and the CNS, which allows a bidirectional communication between the two systems. Its primary role is to monitor and integrate intestinal functions as well as to link, through immune and neuro-endocrine mediators, the emotional and cognitive centers of the brain with peripheral intestinal mechanisms such as immune activation, intestinal permeability, enteric reflex, and entero-endocrine signaling [20]. In this communicating network, the brain affects gut movement, sensory, and secretion functions, and in turn the signals from the gut affect brain function [24]. This relationship is therefore of outmost importance for the maintenance of gut homeostasis, and it has been reported to be also involved in the etiology of several metabolic and mental (psychiatric and neurological) dysfunctions and disorders [21,25].

Different routes of communication between the gut microbiota and the brain have been suggested:

- Through incoming and outgoing branches of the vagus nerve [26], which represents the major modulatory pathway [27].
- Through the generation of metabolites and bioactive peptides (such as short-chain fatty acids) as well as the modulation of transmitters (e.g., serotonin and acetylcholine) by the microbiota [26,27,28].
- Through the secretion of cortisol by the HPA in case of stress, which can affect intestinal motility, integrity, and mucus production, leading to changes in gut microbiota composition. This alteration, in turn, may affect the CNS through the modulation of stress hormones [28].
- Through pro-inflammatory cytokines and chemokines [29].
- Immunity is also critically involved. Specifically, toll-like receptors (TLRs) and peptidoglycans (PGNs) mediate the immune response towards microbes by acting as sensors of microbial components [30,31]. A local immune activation can, throughout different pathways, lead to an immune activation in different organs, including the brain [32]. This low-grade immune activation has been implicated in the pathophysiology of some forms of depression and neurodegenerative disorders such as AD and Parkinson’s disease (PD) [26].

Given this complex interplay, it is not surprising that the gut–brain axis, and therefore the gut microbiota as main component of this crosstalk, directly or indirectly affects neuropsychiatric illnesses [33].

### 2.2. The GMBA in Alzheimer’s Disease: What’s New?

The role of gut microbiota and GMBA in AD is of utmost importance [34]. The composition of the gut bacteria affects dramatically any age-related neurological disorder, such as AD, and mood disorders. Extrinsic factors including diet, lifestyle, or also pro-inflammatory insults, along with intrinsic components including genetic polymorphism, immunity, metabolites, and hormones, profoundly affect the composition of the gut microflora, which in turn produces signaling molecules such as short chain fatty acids (SCFAs), tryptophan, choline, and hormones (such as ghrelin, leptin) in the GI tract able to regulate CNS functions [35]. Aging has a strong impact on gut microbiota composition favoring the development of pro-inflammatory bacteria (such as *Bacillus fragilis*, *Faecalibacterium prausnitzii, Eubacterium rectale*, *Eubacterium hallii*, and *Bacteroides fragilis*) to the detriment of anti-inflammatory bacteria, a condition that induces local systemic inflammation then leading to enhanced permeability of the gastrointestinal tract, an impairment in the blood–brain barrier (BBB), finally promoting neuroinflammation. Indeed, Cattaneo et al. observed such pro-inflammatory bacteria in amyloid-positive patients when compared to healthy subjects [36]. In transgenic mice (mutant human APP) when infected with *Salmonella enterica* and in transgenic *Caenorhabditis elegans* (human Aβ42 peptide) infected with *Candida albicans*, the authors reported a susceptibility to further infections although they died later with respect to wild type animals. Probably, this was due to the antimicrobial activity of Aβ peptide, as the heparin-binding motif of Aβ oligomers make easier the binding to the glycosyl group of the carbohydrate moiety in the bacterial cell wall, so preventing its adhesion to the host cell and the induction of microbial agglutination [37]. Also, bacteria-derived amyloids have been reported to be causative factors for Aβ peptide aggregation in AD. For example, amyloids produced by bacteria such as curli (*E. coli*), TasA (*Bacillus subtilis*), CsgA (*S. Typhimurium*), FapC (*Pseudomonas fluorescens*), phenol soluble modulins (*Staphylococcus aureus*), *etc.*, have been shown to contribute to the development of AD pathology particularly by promoting Aβ oligomers and fibrils formation [38]. 

Besides bacteria-derived amyloids, further components contribute to the onset and pathogenesis of AD. For instance, lipopolysaccharides (LPS) from bacteria inoculated in experimental animals (in the fourth ventricle of the brain) generated a symptomatology very akin to AD [39]. Even the injection of LPS in mice induces elevation of Aβ in the hippocampal area causing cognition defects, thus supporting the role of LPS in amyloid fibrillogenesis [40,41]. When in circulation, LPS has been found to activate the TLR4 pathway, thus triggering immune cells to secrete pro-inflammatory cytokines and IgM/IgA, exacerbating systemic inflammation [42,43,44,45]. In this perspective, gut inflammation may be a cause of AD pathogenesis. 

The relationship among gut microbiome composition, inflammation, further neuroinflammation, and AD onset, is a fundamental matter of debate in AD etiopathogenesis. A certain number of investigations reported the presence of pathogens in the post mortem brains of AD patients [46,47,48,49]. Among them are herpes simplex virus type 1 and bacteria such as *Chlamydophila pneumoniae*, *Borrelia burgdorferi*, or other spirochetes [50,51,52]. Furthermore, a significant increase in the level of *Helicobacter pylori*-specific IgG antibodies, found in the cerebrospinal fluid and in the serum of AD patients, was reported [53]. In this context, novel therapeutic approaches can be envisaged by investigating the crucial role of some gut microbiota compositions leading to AD with the aim of promoting the prevalence of health-associated species, also adjusting both dietary habits and lifestyle, that could help to prevent disease development/progression [54,55,56].

### 2.3. Gut Microbiota Alterations in AD

Intestinal dysbiosis is a condition of microbial imbalance caused by an overgrowth of “bad” bacteria inside the gut, associated with potential negative outcomes such as the incorrect production of essential metabolites or even the genesis of harmful metabolites [14,57]. Although the composition of a “healthy microbiota” has not yet been defined, a balanced environment between the host and microorganisms is known to be essential to carry on the necessary immunological and metabolic functions [58]. Over the past years, dysbiosis has been reported to be implicated in the development of several disorders, such as obesity, diabetes, chronic fatigue syndrome, intestinal bowel syndrome, cancer, autoimmune diseases, depression, anxiety, PD, multiple sclerosis, amyotrophic lateral sclerosis, and other neuropsychiatric disorders [59,60,61,62,63,64,65,66,67,68,69]. Recently, many studies have shown that gut microbiota alterations directly influence cognitive decline, actively participating in AD pathogenesis and progression [36,70,71,72,73]. Generally, AD patients are often characterized by a decreased gut microbial diversity, with a significant shift in favor of pro-inflammatory taxa at the expense of the more beneficial anti-inflammatory ones, similar to what has been observed in both mouse and human aging [25,36,70,71,72,73,74,75]. For example, when fecal microbiota 16S rRNA sequencing was performed on 97 individuals [33 AD, 32 MCI (mild cognitive impaired) and 32 controls], a significant decrease in *Firmicute*s was accompanied with a higher *Proteobacteria, Gammaproteobacteria,* and *Enterobacteria* abundance in patients with neurodegeneration compared to healthy subjects. Interestingly, a pronounced difference in *Enterobacteriaceae* has been reported also between MCI and AD patients, thus indicating a progressive change in the gut microbiota composition during disease progression [72]. Similarly, Vogt et al. detected a significant dampen in *Firmicutes* and *Bifidobacteria* in the fecal samples of AD patients, and this decrease was counterbalanced by the overgrowth of *Bacteroidetes* species in the same individuals [70]. Alterations in the gut microbiota composition during neurodegeneration has also been reported by Zhuang et al. when comparing 43 AD patients with age- and sex-matched controls: enriched *Bacteroidetes* and decreased *Actinobacteria* at the phylum level were paralleled by enhanced *Ruminococcaceae*, *Enterococcaceae,* and *Lactobacillaceae,* together with less *Lanchnospiraceae*, *Bacteroidaceae,* and *Veillonellaceae* at the family level [76]. However, in contrast with this evidence, lower *Bacteroides*, *Lachnospira,* and *Ruminiclostridium* and higher levels of *Prevotella* have been reported in another study [77]. Although reductive, this discrepancy might be at least in part explained by the different geographical origin of the participants, since regional identity may strongly affect gut microbiota composition, as well as other comorbidities [78]. In this respect, larger studies are certainly needed to establish standard and reproducible inclusion criteria, possibly excluding also the possible confounding effect of other comorbidities.

A growing body of evidence indicates that gut microbiota dysfunctions are involved in the early disease stages of AD pathogenesis, enhancing immuno-senescence, oxidative stress, cytokine secretion, and neuroinflammation [79]. In this respect, Cattaneo et al. report that patients with AD show an increase in pro-inflammatory endobacteria species of *Escherichia/Shigella* and a decrease in the anti-inflammatory taxon *E. rectale*, and that this microbiota alteration is associated with amyloidosis and peripheral inflammation [36]. Moreover, when stool samples were collected from 108 nursing home elders and analyzed with metagenomic sequencing, a decline in butyrate-synthetizing bacteria was paralleled by a rise in pro-inflammatory taxa in AD elders, thus possibly exacerbating local and systemic inflammation [71]. Interestingly, these data have been correlated with low levels of expression of the P-glycoprotein, an essential molecule required for intestinal homeostasis, therefore indicating a clear nexus between microbiome dysregulation and intestinal inflammation [71]. These results further support the concept that changes in gut microbiota composition also reflect in alterations in intestinal function. Indeed, differences in gut microbiota population may influence tryptophan and serotonin levels in the body and may affect the synthesis of some key molecules useful for the brain, such as dopamine, norepinephrine, and brain-derived neurotrophic factor (BDNF) [80,81,82]. As mentioned, another beneficial role exerted by the gut microbiota is the production of SCFAs, including butyrate, propionate, and acetate, essential for energy production, gut epithelia homeostasis, and immune regulation [83]. When their production is altered as a consequence of dysbiosis, Aß plaques deposition, metabolic dysfunctions, and microglia dysregulation is favored, thus promoting cognitive decline [84,85,86]. Moreover, a decrease in butyrate-producing bacteria, as reported in AD, has been linked to T cell imbalance, epithelial barrier leakage (so called “leaky gut”), and increased bacterial translocation [80,87,88]. Consequently, circulating Gram negative endobacteria-derived LPS, also known as metabolic endotoxemia, triggers systemic inflammation via TLR4 and promotes BBB disruption, thus fostering neuroinflammation. [89,90]. Intestinal dysbiosis can also contribute to the increase of harmful substances such as amyloid and trimethylamine N-oxide (TMAO). TMAO is a microbial metabolite that has been recently implicated in increased formation of beta amyloid, peripheral immune response activation, enhanced oxidative stress, platelet hyperactivity, intestinal mucosal barrier dysfunction, and BBB permeability, thus promoting the consequent passage of bile acids produced by bacteria and cholesterol in the brain [91,92,93,94,95]. Finally, the ability of some endobacteria to produce gaseotransmitter molecules, such as nitric oxide (NO), hydrogen (H_2_), ammonia (NH_3_), methane (CH_4_), and hydrogen sulfide (H_2_S) seems to be fundamental for the proper neuronal function, and its alteration participates to AD pathogenesis [96,97]. Overall, these data indicate that the dialogue between gut microbiota and brain is much more complicated than previously thought, and only its entire understanding can provide insights into new diagnostic and therapeutic interventions.

### 2.4. Gut Microbiota-Based AD Biomarkers

One of the major concerns in AD research is to find predictive, sensitive, non-invasive accurate, and accessible biomarkers for early disease diagnosis [98,99]. Although many studies focus on fluid biomarkers for early disease detection, we are still far from having found an effective and consistent assay to be used in the clinical practice [100]. As mentioned above, the gut microbiota has emerged as a key player in regulating both physiological and non-physiological conditions, thus gut microbiota-related biomarkers may represent a promising alternative/complementary tool to assess disease conditions [101]. Indeed, although initially hypothesized for gastrointestinal disorders [102], gut microbiome-derived biomarkers have also been considered for psychological and neurodegenerative diseases (i.e., bipolar disorder, multiple Sclerosis, and PD), reporting powerful predictivity and differential diagnosis ability [103,104,105]. Regarding AD, promising results have recently been obtained, and Table 1 summarizes the main findings [72,73,106,107,108,109,110,111,112,113,114,115] (Table 1). Whilst species of *Prevotella* and *Helicobacter* have been shown to be significantly different between APP/PS1 transgenic mice and controls, *Actinobacteria* and TM7 phylum seem to be more accurate in diagnosing AD when using the triple transgenic mouse model [106,107,109]. Changes in beta diversity and variations in circulating metabolites involved in inflammatory pathways and metabolism of nucleotides, lipids, and sugars (i.e., glutamate, hypoxanthine, thymine, hexanoyl-CoA, and leukotrienes) have also been considered in the same studies, showing promising results [106,107]. Remarkably, when the gut microbiota of APP/PS1 mice at different ages was compared to matched controls, huge shifts in the abundance of the families *Proteobacteriaceae, Verrucomicrobiaceae, Bifidobacteriaceae, Erysipelotrichaceae, Prevotellaceae, Bacteroidaceae,* and *Rikenellaceae* could be detected far before any plaque deposition in the brain, suggesting a great potentiality for early diagnosis [110]. Although nowadays, mice clearly represent the most used animal model, some evidence obtained with *Drosophila melanogaster* indicate *Wolbachia* as a potential AD biomarker, while *Stenotrophomonas* appears to exert a beneficial role in preventing neurodegeneration [111].

In humans, when a cohort of individuals with AD and/or MCI were compared to healthy controls, significant differences in microbial diversity and in the fecal and blood abundance of 11 genera were observed [112,113]. Importantly, Li et al. report no major variation in the analyzed gut microbiota biomarkers between MCI and AD groups, suggesting a better ability in early detection rather than in clinical progression monitoring [113]. Similarly, cerebrospinal fluid levels of the gut microbiome-dependent metabolite TMAO were not different in AD compared to MCI patients, although significantly higher than controls [114]. On the contrary, other studies report the capability of some biomarkers (i.e., *Enterobacteriaceae*, SCFAs, and indole-3-pyruvic acid abundance) to clearly differentially diagnose between a mild symptomatic disease (MCI) and a more advanced stage, thus leaving the debate open [72,115]. Other suggestions might come from the evidence of a progressive shift from *Faecalibacterium* to *Bifidobacterium* genera in AD, thus offering the possibility to follow the ratio of butyrate/lactate producing genera as a disease marker of neurodegeneration [73]. Although the data are still limited, it would be interesting to see the results of the currently undergoing Emory Healthy Aging and Emory Healthy Brain Studies, aimed at following 50–75 years old individuals (without AD or any other cognitive impairment), to identify early disease biomarkers, comprised the gut microbiome ones [116]. Biosignatures from the gut microbiota might also be exploited for patients’ stratification and therapy in clinical trials aimed at applying precision medicine in AD treatment, but the information remains for now limited [117]. Finally, although our review focuses on the gut microbiota, it is important to mention that oral microbiota has been implicated in AD pathogenesis and might also represent a source of novel salivary biomarkers in AD, possibly in a combinatorial approach with the gut microbiota ones [118,119,120,121,122].

Although these preliminary data may appear promising, several limitations still exist and must be accounted. First, when looking for a new biomarker, large cohorts should always be preferred over smaller ones, and the evidence obtained should be confirmed on a validating group [100]. Secondly, since the gut microbiome composition changes widely according to nationality, lifestyle, and dietary habits, it is not often easy to distinguish between real evidence and confounding factors, thus questioning the relevance of the results [123]. Additionally, the importance of age- and gender-matched control groups should not be underestimated, and the respective cohorts should be designed accordingly [100]. To partially solve these limitations, a combination of different biomarkers could be adopted. For example, Zhang et al. report how gut microbiota composition, serum miRNAs and dietary quality scores can be used together to improve reproducibility and consistency [112]. In this respect, it would be interesting to investigate whether SCFAs, in combination with other fluid biomarkers, might prove effective in disease diagnosis and clinical monitoring, as some evidence already suggested [108].

Overall, although there are still some limitations, these data indicate that gut microbiota-based biomarkers might represent an alternative and/or an integration to the existing ones and should encourage scientists to plan larger investigations in humans.

### 2.5. Prebiotics

Prebiotics are non-digestible organic substances (i.e., short-chain carbohydrates) capable of selectively stimulating the growth and/or activity of one or a limited number of beneficial bacteria present in the gut [124]. Being used as food from the gut microbiota, they stimulate the production of SCFAs, thus influencing both gastrointestinal and extra-intestinal functionality [125]. A growing body of evidence suggests their potentiality as adjuvant therapy in different neurological and psychiatric conditions, such as anxiety, depression, and PD [126]. Recently, some studies are also considering the use of prebiotics for AD prevention/therapy, with promising results [124,126,127,128,129,130,131,132,133,134,135,136]. For example, yeast beta glucans administration to mouse models of AD proved effective in re-establishing the balance between pro-inflammatory and anti-inflammatory gut microbiome species, promoting SCFAs production and limiting neuroinflammation and insulin resistance [127]. Reduced neuroinflammation and improved short-term memory and cognitive ability in mice resembling AD features were also reported upon pre-treatment with lactulose and melibiose, two trehalose analogues, possibly via enhanced autophagy function [129]. Furthermore, 5xFAD mice fed for eight weeks with mannan oligosaccharide were shown capable of favoring the growth of *Lactobacillus* species and decreasing *Helicobacter* abundance, therefore preventing LPS leakage and intestinal epithelial barrier and BBB dysfunctions [130]. Interestingly, this prebiotic-driven reshaping of the gut microbiota was also accompanied by reduced Aβ accumulation in different brain areas (i.e., cortex, hippocampus, and amygdala), re-established redox homeostasis, and increased butyrate levels [130]. Similar results were also obtained in both rats and mice models of AD via oral administration of *Marinda officinalis*-derived oligosaccharides, reporting improved memory and learning ability, together with a decrease in plaque formation, oxidative stress, and overall inflammation [134,135]. Although the mechanism of action of the above-mentioned prebiotics is not totally clear, the capability of these molecules to sustain gut microbiota diversity and stability might be at the basis of these improvements [127,132,134]. This hypothesis is reinforced by recent evidence showing that a combination of probiotics and prebiotics (so-called synbiotics) seems to be more effective in increasing neurogenesis and reducing local and systemic inflammation compared to prebiotics alone [132]. 

Regarding humans, data on a large multi-ethnic longitudinal study comprising 1837 elderly people with no evidence of neurodegeneration have shown that daily administration of fructan, a well-known prebiotic, reduces the risk of AD development, confirming the previous evidence in mice [131]. However, despite this study being conducted normalizing for age, gender, recruitment time, ethnicity, daily caloric intake, education, and APOE genotype, other authors point out that the evidence for the use of prebiotics in the clinical practice still lacks robustness [133]. Altogether, these data suggest that prebiotics may be helpful as preventative/adjuvant therapy for AD, but more human clinical trials are needed before drawing any conclusion. 

### 2.6. Probiotics

In 1965, Lilly and Stillwell introduced for the first time in the literature, the term “probiotics”, defining them as “living microorganisms with a low or zero pathogenicity that provide beneficial effects on the health of the host” [137]. Studies on human and animal models have shown that probiotics can modulate intestinal ecosystem homeostasis, regulate intestinal epithelial functions by helping to maintain the epithelial barrier, producing SCFAs, supporting cell survival, enhancing protective immune response, and inhibiting the production of pro-inflammatory cytokines [83,138,139,140,141,142,143,144,145,146,147]. Many of these responses arise from the regulation of specific intracellular signaling ways by probiotics, such as mitogen-activated protein kinases (MAPK) and nuclear factor (NF) -κB in intestinal epithelial cells [83,138,139,140,141,142,143,144,145,146,147]. Probiotic bacteria, through the modulation of the intestinal microbial ecosystem, have shown capable of playing an important role in immune response regulation by Th1, Th2, Th17, Treg cells, and NK and B cells stimulation [148]. Several studies have also confirmed the anti-inflammatory capacity of specific probiotics, by modulating the cytokine network and the macrophage tissue pattern, to reduce the mucosal inflammatory process and modulate the local immune response [149].

Probiotics can also modulate the gut–brain axis. The so-called psychobiotics, a new class of probiotics with potential applications in the treatment of psychiatric diseases, are able to modulate the bidirectional communication between brain and gut through the modulation of neurotransmitters and proteins, including gamma-aminobutyric acid, serotonin, glutamate, and the brain-derived neurotrophic factor, which play important roles for the functionality of our central nervous system, mood, cognitive functions, learning and memory processes [150,151,152,153]. The administration of a probiotic mixture modified the gut microbiota in an animal model of AD by increasing *Actinobacteria* and *Bacteroides* with a significant impact on the enhancement of long-term memory, inflammation, and neural plasticity [154]. Mitochondrial dysfunction, excessive production of reactive oxygen species, and increased apoptosis have been implicated in the pathogenesis of AD. In this respect, several studies have highlighted the role of superoxide anion, hydroxyl radical, hydrogen peroxide, and nitric oxide in neurodegeneration mediated by oxidative stress in AD [155,156]. Recently, a study on transgenic AD mice demonstrates that the administration of a probiotic formulation (SLAB51) significantly reduces oxidative stress by inducing SIRT-1-dependent mechanisms [157]. In addition, the probiotic integration of a multispecies mixture of *Lactobacillus* and *Bifidobacterium* has proven capable of modifying specific brain metabolites such as γ-aminobutyric acid and glutamate [158]. Immune response and neural inflammation were also suppressed after probiotic integration with short A1 strain of *Bifidobacterium* [159]. Furthermore, the integration of *L. acidophilus*, *L. fermentum*, *B. lactis*, and *B. longum* improved learning disability and oxidative stress of rats subjected to intra-hippocampal injection of Aβ1-42 [160].

Although these studies on animal models show that probiotics may play an important role in two-way communication between gut and brain and support the potential role of probiotics in improving cognitive health, the results of clinical studies in subjects with AD or MCI are controversial.

In a recent randomized, double-blind, clinically controlled trial, 60 AD patients were divided into two groups and administered milk (control group) or probiotics (probiotic group). After 12 weeks of daily administration of 200 mL of a mixture of *Lactobacillus acidophilus*, *Lactobacillus casei*, *Bifidobacterium bifidum*, and *Lactobacillus fermentum*, a significant improvement in the mini-mental state exam (MMSE) score was reported compared to controls (*p* < 0.001). Changes in plasma malondialdehyde, serum C-reactive protein, beta cells function, serum triglycerides, and differences in the quantitative control index of insulin sensitivity were also improved in the individuals receiving the probiotic mixture [161]. Similarly, data from another meta-analysis report a significant amelioration in cognition and a consistent reduction in post-intervention levels of malondialdehyde and high sensitivity C-reactive protein in subjects receiving probiotics compared to controls [162]. Although these results indicate potential benefit of probiotics in the management of patients with AD, other studies show contrary data. For example, in a recently published randomized, double-blind, placebo-controlled clinical trial, AD patients (between 65 and 90 years old) were supplemented with placebo (control group, *n* = 23, 13 females and 10 males) or a probiotic mixture (probiotic groups, *n* = 25, 18 females and 7 males). Two different probiotic capsules were used in this study: one containing *Lactobacillus fermentum*, *Lactobacillus plantarum,* and *Bifidobacterium lactis*, and one containing *Lactobacillus acidophilus*, *Bifidobacterium bifidum*, and *Bifidobacterium longum*. After 12 weeks of alternate day administration, the levels of proinflammatory (TNF-α and IL-6) and anti-inflammatory (IL-10) cytokines, as well as the levels of oxidizing (MDA and 8-OHdG) and antioxidants factors (TAC, GSH), were not significantly changed between the two groups. Of note, no improvements in cognitive functions were reported in the probiotic group compared to the placebo one, suggesting insensitivity to probiotic supplementation for severe AD patients [163].

In conclusion, even if there are several studies that show the influence of gut microbiota in neurological and psychiatric pathologies, the mechanisms of action and the effects of probiotics rest largely unknown, and several gaps and inconsistences remain. Therefore, human studies need to be further developed and need to include analysis of the gut microbiota composition in specific populations of patients by identifying probiotic bacteria strains able to significantly affect gut–brain axis and assess their safe use.

### 2.7. Diet

Diet is a rapid and direct way of modifying the gut microbiota composition and function, reducing inflammation, and helping in eubiosis maintenance [123,164,165]. Given the evidence of association between neuropsychiatric conditions and gut microbiota dysregulation, it is worth speculating that dietary interventions could represent effective candidates for preventing and delaying the pathogenesis and progression of AD [166,167,168,169,170,171,172,173,174,175,176,177,178,179,180,181,182,183,184,185,186] (Table 2). Here, we present some of the most promising dietary therapies proposed in the literature, with a particular focus on Mediterranean and ketogenic diets.

#### 2.7.1. Mediterranean, DASH (Dietary Approaches to Stop Hypertension), and MIND (Mediterranean-DASH Intervention for Neurodegenerative Delay)

The renowned and ancient Mediterranean diet (Medi), rich in vegetables, fruit, whole grains, nuts, olive oil, moderate consumption of fish and poultry and limited consumption of red meat and sweets, have been extensively described for their protective role against non-communicable diseases [187]. DASH diet (Dietary Approaches to Stop Hypertension), designed for hypertension treatment, overlaps the Medi diet in composition, with more attention on salt introduction (less than 2.4 g/day) [188]. Similarly, MIND diet (Mediterranean-DASH Intervention for Neurodegenerative Delay) is a combination of both, DASH and Medi, specifically developed to delay neurodegeneration. Besides being rich in fruits, vegetables and legumes, the MIND includes the consumption of single dietary components, i.e., green leafy vegetables and berries, which have displayed a superior effect against cognitive impairment and decline compared to other vegetables and fruits [189].

Although Randomized Clinical trials (RCT) and Observational Cohort Studies (ObS) have been conducted to unravel the potential therapeutic effect of Medi, DASH, and MIND in AD, the results are still unclear [166]. Two large RCT conducted in Spain in 2013 and 2015 have demonstrated a positive correlation between ‘Medi diet plus olive oil’ or ‘Medi diet plus nuts’ with cognitive performance [190,191]. More recently, an additional RCT study also associated Medi diet with improved cognition [192]. However, differential results from another RCT did not show any significant association [193]. Further narrative, systematic reviews, and meta-analyses have evidenced the protective and promising therapeutic role of Medi diet in AD disease, confirming its ability to hinder cognitive impairment [167,169]. Generally, any dietary pattern rich in fruits, vegetables, and legumes and poor in saturated fats and sweets seems to provide protective effects [194]. Similarly, results presented by Barbaresko et al. on 20 systematic reviews and meta-analyses, highlighted the benefits of the Medi diet as a protective factor for AD [195].

So far, besides promising results for Medi diet, the role of DASH diet in AD prevention and therapy is still unveiled [168], and more studies should be carried out before driving any conclusion. Moreover, standard tools for assessing food intake and cognitive decline are needed to state which dietary pattern might be the most effective in protecting and delaying the onset of neurodegenerative diseases, and to ensure reproducibility [166].

Concerning MIND diet, Morris at al. were the first to show that a moderate adherence to this dietary habit slows cognitive decline compared to a moderate adherence to Medi and DASH diets; however, they have also confirmed that a high adherence to Medi and DASH diets can reduce AD risk [189]. Potential neuroprotective mechanisms shared by those dietary regimes are the presence of antioxidant and anti-inflammatory compounds, which contribute to a reduction in brain inflammation and oxidative stress, high abundances of fibers, vitamin C, beta-carotene, and folate, which lead to a better brain integrity and increase in brain tissue volume [196,197]. Also, the scarcity in saturated and trans fatty acids can reduce BBB dysfunction and amyloid aggregation [183,198,199].

A significant improvement in cognition was also reported among older adults following the MIND diet, confirming the effectiveness of this approach [170]. Despite those findings, the lack of evidence on the correlation between MIND diet and brain-related mechanisms, and given the similarities with the Medi and DASH diets in terms of nutrients composition, MIND diet cannot be disclosed as more proactive than Medi and DASH diets.

On the whole, as previously mentioned, the protective and potential therapeutic effect of Medi (and similar diets) might be based on the consumption of much food rich in vitamins and polyphenols, i.e., fruits, vegetables, legumes and whole grains, a moderate amount of fish, and less meat and food rich in trans and saturated fats. Regarding meat, so far, the majority of the studies did not report any significant association with cognitive impairment or decline [177]. Differently, fish intake is inversely associated with AD—likely related to omega-3 (EPA/DHA) contents [195]. Interestingly, the regular consumption of fish up to two portions per week seems to be more protective than EPA/DHA supplementation [184]. Even though many studies are supporting the protective effect of unsaturated fatty acids EPA/DHA, their role in the brain is still under debate [200,201,202,203,204]. Medi diet is also connected to an improved lipid profile. Overall, lipid dysregulation might contribute to AD pathogenesis, enhancing synaptic loss, BBB dysfunction, mitochondrial disruption, oxidative stress, and inflammation [198,205]. Indeed, in large longitudinal studies, high levels of triglycerides and cholesterol in the serum are significantly associated with cognitive impairment [178]. Again, a recent cross-sectional study with 689 participants including AD and healthy patients, revealed that reduced levels of triglycerides were related to better cognitive performance and a reduction in brain dysfunction and atrophy [206]. In conclusion, even if the interplay between dietary lipids and AD pathogenesis is not straightforward, Medi diet with consequent improvement in lipid dysregulation through dietary changes is strongly recommended.

Dietary regimens based on a daily integration of the essential nutrients and vitamins are also of interest, but the data remain limited. Cross-sectional and longitudinal cohort studies on vitamins C and E consumption showed promising effects in reducing cognitive decline, but no difference has been identified in intervention trials [182]. Similarly, low levels of vitamin D in the serum seem to be associated with an increased risk of cognitive decline, but its supplementation did not provide any difference [182]. Vitamin B (folic acid, pyridoxine, and cobalamin) consumptions lead to ambiguous results, with only a few RTC displaying beneficial effects in slowing the cognitive decline [182]. Finally, even though vitamin A supplementation might reduce the risk of cognitive decline, there are not enough consistent data to confirm its protective and therapeutic effect in AD [182]. Overall, it seems that some vitamin supplementation might delay the progression of AD and dementia; nonetheless, due to the lack of statistically significant results and limited scientific evidence analyzing the role of vitamins in older adults [167,185], it is not possible, at least until now, to point out their specific protective and therapeutic effects in AD.

Besides micronutrients and omega-3, further nutritional formula including ginseng, inositol, and coconut oil have been recently studied as potential therapy in AD patients, but the effects are inconclusive [176].

Polyphenols are receiving growing interest in AD research due to their antioxidant, anti-inflammatory, and neurotrophic properties supported by preclinical evidence. Nonetheless, so far, there is no conclusive evidence on the association between polyphenols and AD in humans. On 24 RCT conducted on AD patients exposed to polyphenols (mainly flavonoids), only 12 have shown a reduction in cognitive decline [181]. Again, further trials carried out in people with mild cognitive impairment consuming grape juice or blueberries rich in polyphenols showed minimal benefits in memory or no significant results [179,180]. A polyphenol that might contribute to neuroprotection is resveratrol. This phenolic compound promotes synthesis of glutamate receptors, enhances synaptic transmission, activates SIRT1, exerts antioxidant and anti-inflammatory actions [186,207]. Results from in vitro and in vivo (mice and rats) studies underscored resveratrol as a potential treatment for AD; however, its effectiveness is only partially understood in humans [208]. Although some research groups have performed trials in humans to test the potential protective effect of resveratrol, results have failed to demonstrate a positive correlation. The lack of a substantial number of clinical trials and issues related to clinical applications, e.g., dosage, bioavailability, side effects, etc., emphasize the need of further investigation [186].

#### 2.7.2. Ketogenic Diet

Ketogenic diet (KD) is a nutritional program rich in fats and low in carbohydrates and proteins (ideally, 90% fat, 4% carbohydrates, 6% proteins) developed in the early 1990s as a treatment for epilepsy, with numerous studies consistently supporting its effectiveness [209]. Recently, the application of KD as potential treatment for other neurological diseases, such as PD and AD, has been investigated in vitro and in vivo [210,211,212]. The sugar-shortage leads the body to break down and oxidate fats with the production of ketone bodies, used as an alternative energy-substrate to glucose by many organs, including the brain [213]. In mice models, ketone bodies influence neurotransmission, channels modulation, increase BDNF, reduce neuroinflammation and oxidative stress, improve mitochondrial functions, reduce amyloid accumulation, and improve learning and memory abilities [213,214,215,216]. In humans, results from RCT reported that KD might be beneficial in people with mild cognitive impairment or AD [171,172]. Similar to KD in terms of mechanisms (i.e., ketone bodies production), medium-chain triglyceride (MCT) diet/supplementation and the modified Atkins diet are effective in counteracting cognitive decline in AD, symptoms such as fatigue and daytime sleepiness in PD, epileptic seizures and mood swings in depression [173,174,175,217,218]. Moreover, the modified Atkins diet, which does not restrict protein intake as the KD protocol, allows a much more nutritional flexibility than classic KD. Indeed, overall, dietary patterns that lead to ketone bodies production seem to represent a promising therapy for AD, but more investigation to unveil protective mechanisms in humans and adverse aspects is needed—including the lack of flexibility and variability of the alimentary regimen easily leading to a drop-out, the scarcity of plant-based food rich in vitamins, and other antioxidant compounds [219].

Even if further well-designed human clinical trials are needed to better understand the role of diet for the prevention and treatment of AD, up to now, most of the diet-related beneficial effects in AD patients seem to be in favor of the Medi diet. Vitamins and other phenolic compounds might represent potential boosts for AD patients.

#### 2.7.3. The Role of Diet in AD Mediated through Gut Microbiota

Diet is the most impactful modulator of gut microbiota across lifespan. Considering that, as previously mentioned, AD is associated with changes in microbiota composition, it is reasonable to assume that dietary interventions, and the related gut microbiota composition shifts, might constitute a future complementary tool to prevent or manage dementia. However, the proof of a cause–effect relationship among gut microbiota, diet, and neurodegeneration is very poor, with a low number of clinical studies analyzing the interplay among those elements.

Current evidence shows that the Medi diet beneficially impacts the gut microbiota composition in elderly adults reducing frailty [220,221]. The abundance of specific “protective” taxa (e.g., *Faecalibacterium* or *Roseburia*) was positively associated with improved cognitive function and negatively associated with pro-inflammatory markers, possibly related to an increase in SCFAs [165]. Medi diet is rich in polyphenols, and emerging evidence supports the beneficial role of polyphenols in preventing and/or ameliorating AD progression, reducing plaques formation and protecting blood brain barrier disruption [222]. Wine polyphenols, for instance, may lead to an increase in *Bacteroides*, *Bifidobacteria,* and *Lactobacilli*, re-storing a ‘healthy’ microflora composition in AD patients [223].

Improvement in the microbiota profile as a result of KD was initially showed in preclinical animal models, where mice subjected to KD diet displayed an increase in *Akkermansia* and *Lactobacillus* and a parallel enhancement in vascular brain function [224]. An interventional study, where MCI patients followed a modified Medi-KD, demonstrated that dietary regimen positively affects the gut microbiota composition, with an increase in the abundance of *Enterobacteriaceae*, *Akkermansia*, *Christensenellaceae,* and in SCFAs production, with a consequent improvement in cognitive symptoms [225]. Nonetheless, while a recent study in an animal model of AD revealed that KD might exacerbate gut dysbiosis, a diet rich in carbohydrates seemed to improve the microbiota profile with an increase in *Bacteroidetes* and a reduction in *Proteobacteria* [226,227]. Dietary patterns that allow not-refined carbohydrates consumption, but still lead to ketone bodies production, e.g., intermitting fasting, might be a promising protective dietary strategy for dementia [226].

Decoding the interplay between microbiota and diet in neurogenerative disease patients seem to be promising; however, all the multi-faceted aspects of dietary patterns on human health should be examined in depth, considering the body as a superorganism, made of human and microbial cells.

### 2.8. Fecal Microbiota Transplantation

Fecal microbiota transplantation (FMT) is a procedure where a solution of fecal material from a donor is transferred (through colonoscopy, nasogastric tube, or oral pills) into the intestinal tract of a recipient, aimed at directly changing the gut microbiota composition [227]. Reprogramming the gut microbiota *eubiosis* by FMT has been already used to successfully treat *C. difficile* infections and could be an innovative therapy for various neurological diseases in an imminent future [228]. So far, most of the limited number of studies have been conducted in mice/rats, with promising but not conclusive results (Table 3) [10,229,230,231,232,233,234,235,236,237,238,239].

Impaired neurogenesis, decreased BDNF expression, increased memory impairment, enhanced circulating pro-inflammatory cytokines, and Aβ plaques deposition were detected when feces from AD-model donor mice were transplanted in healthy mice [231,233]. Moreover, FMT from senescence-accelerated mice or from senescence-accelerated-resistant mice into germ-free (GF) mice revealed significant differences on behaviors, cognitive performance, and gut microbiota composition, with a better profile in recipient mice receiving the microbiota from senescence-accelerated-resistant donors compared to senescence-accelerated mice [236]. Similarly, GF mice receiving fecal material from APPPS1 transgenic mice developing cerebral Aβ-deposition showed an increase in plaques formation [234,235]. When FMT was carried out from an AD patient into GF mice, accelerated cognitive decline and a decrease in microbiota-derived metabolites important for the nervous system function were reported [235].

Successfully, researchers confirmed that interventions aimed at manipulating gut microbiota influence brain disorders. Indeed, transplanting healthy fecal microbiota from wild-type mice to mouse models of AD documented a decrease in cognitive impairment, amyloid accumulation, and circulating levels of pro-inflammatory markers [10]. Improved cognition, reduced amyloid accumulation and tau expression, enhanced synaptic plasticity, and increased SCFAs-producing gut endobacteria were also confirmed in another study [232]. Dodiya et al. reported the effectiveness of FMT in restoring microbiota composition in the APP/PS1 transgenic mouse model of AD, improving microglia and Aβ deposition profile [237].

Regarding humans, only two case-studies showing promising results have been conducted so far [229,230]. Hazan et al. demonstrated an improvement in AD symptoms (cognitive function, memory, and mood) in a 82-year-old man after FMT from a 85-year-old woman (recipient’s wife) [229]. A second case-study, involving a 90-year-old woman with AD and severe *C. difficile* infection who received FMT from a 27-year-old healthy man, also showed an improvement in cognitive function, microbiota diversity, and SCFAs production [230].

Despite the potential application of FMT in AD treatment, several limitations still exist. Standardization of the therapeutic protocols, timings and length of administration, short and term risks, and inclusion criteria are all points that should be considered and addressed [68,240,241,242,243,244].

In conclusion, although the promising results obtained in mice certainly prove that the gut microbiota is involved in the pathogenesis and progression of neurological diseases, more human studies are needed before pointing out FMT as an AD complementary therapy.

## 3. Conclusions

AD is a neurodegenerative disorder, often occurring in the elderly, which has a fundamental causative source in the impairments in the GMBA. Recent data, which are to be further deepened and improved in any investigation planning, reported to date a close relationship between gut microbiota composition (then affected by nutritional habits) and AD onset, usually derived from neuroinflammation caused by bacteria products or bacterial brain migration, a circumstance that normally occurs to contribute to the regulation of brain synaptogenesis and development, besides mood and cognition evolution. Given the close cross talk between gut bacteria and brain, here we reviewed that gut microbiota dysregulations, often reported in AD patients, can be exploited to investigate both new diagnostic and therapeutic approaches for this devastating disease. However, despite promising results have been published, more research is needed to limit interstudy inconsistencies and enhances reproducibility before considering a clinical application. 

## Figures and Tables

**Table 1:** Gut microbiota-based biomarkers for AD.

Ref.	Journal	Study Cohort and Design	Analysis Performed	Results	Biomarker/s Proposed
Yan et al., 2021 [106]	Front. Aging Neurosci.	APP/PS1 transgenic mice (8 months old, *n* = 7) receiving fasudil (ADF group) or saline (ADNS group) were compared to age- and gender- matched WT mice	Fecal metagenomic and metabolites	↑ *Firmicutes*/*Bacteroidetes* in ADNS compared to WT ↓ *Firmicutes*/*Bacteroidetes* in ADF compared to WT ↑Metabolites involved in metabolism of nucleotides, lipids, sugars and inflammation	*s_Prevotella_sp_CAG873* as ADF biomarker *s_Helicobacter_typhlonius* and *s_Helicobacter_sp_MIT_03-1616* as ADNS biomarkers Glutamate, hypoxanthine, thymine, hexanoyl-CoA, and leukotrienes in ADF or ADNS
Bello-Medina et al., 2021 [107]	Front. Neurosci.	Mice 3xTg-AD 3 and 5 month-old (*n* = 10 females and *n* = 10 males) compared to matched controls	Fecal sample collection, α and β diversity, LDA and LEfSe	↓ *Actinobacteria* and TM7 in 3xTg-AD compared to controls at 3 month-old ≠ β diversity in female and male 3xTg-AD mice compared to controls	*Actinobacteria* and TM7 phylum alterations β diversity changes Increase in the bacteria families and genera: *Gemella, Allobacullum* and *Selenomonas*
Gu et al., 2021 [108]	Alzheimers Res. Ther.	APP/PS1 transgenic mice (*n* = 11) were compared to WT	16S rRNA sequencing of the gut microbiome and integrated metabolomics	↓ SCFA-producing bacteria (i.e., *Parasutterella* and *Blautia*) in APP/PS1 mice compared to controls ↑ Gut dysbiosis in APP/PS1 mice compared to controls ↑ *Firmicutes*/*Bacteroidetes* in APP/PS1 compared to WT	Inflammatory factors (IL-6 and INF-γ), phosphatidylcholines and SCFA-producing bacteria as combinatorial biomarker for AD
Shen et al., 2017 [109]	J. Alzheimers Dis.	APP/PS1 transgenic mice were compared to WT	16S rRNA sequencing	↓ Gut microbiota diversity in APP/PS1 mice compared to controls ↓ *Prevotella* in APP/PS1 compared to controls ↑ *Helicobacteraceae* and *Desulfovibrionaceae* in APP/PS1 compared to controls	Gut microbiota signature in AD and controls
Chen et al., 2020 [110]	Biomed. Res. Int.	APP/PS1 transgenic mice were compared to WT controls (*n* = 14–24 at 1–2–3–9 months and *n* = 31–34 at 6 months)	16S rRNA sequencing from fecal samples	↑ *Proteobacteriaceae*, *Verrucomicrobiaceae*, *Bifidobacteriaceae*, *Erysipelotrichaceae* and *Prevotellaceae* in APP/PS1 mice ↓ *Bacteroidaceae* and *Rikenellaceae* in APP/PS1 mice	Changes in gut microbiota composition precede plaque deposition: early biomarker
Tan et al., 2020 111]	Benef. Microbes	*Drosophila melanogaster* AD model compared to WT controls	Gut microbiota composition analysis	↑*Wolbachia* in AD flies compared to controls ↓Gut microbiota diversity in AD flies compared to controls	*Wolbachia* as a potential biomarker for AD *Stenotrophomonas* negatively correlated with neurodegeneration
Zhang et al., 2021 [112]	Am. J. Clin. Nutr.	Humans: 75 MCI individuals and 52 heathy controls	Changes in gut microbiota and serum miRNA expression	↓ Microbial diversity, *Faecalibacterium*, *Ruminococcaceae*, *Alipstes* in MCI compared to controls ↑Proteobacteria and *Gammaproteobacteria* in MCI compared to controls	Differential gut microbiota composition, diet quality scores and serum miRNA as combinatorial biomarker for MCI patients
Li et al., 2019 [113]	Alzheimers Dement.	Humans: AD patients (*n* = 30), MCI patients (*n* = 30), heathy controls (*n* = 30).	Analysis of microbiota community in the faeces and blood via 16S rRNA sequencing	↓ Microbial diversity in AD and MCI compared to controls ≠ 11 genera in the feces and in the blood between AD/MCI and controls = Genera in the blood and feces between AD and MCI	Changes in gut microbiota as early diagnosis in AD
Liu et al., 2019 [72]	Brain Behav. Immun.	Humans: AD patients (*n* = 33), MCI patients (*n* = 32) and healthy controls (*n* = 32)	16S rRNA MiSeq sequencing and phylogenetic investigation of communities by recontruction of unobserved states	↓ Microbial diversity in AD compared to MCI and controls ↓ *Firmicutes* in AD compared to controls ↑ *Proteobacteria* in AD compared to controls ↑ *Gammaproteobacteria*, *Enterobacteriales* and *Enterobacteriaceae* in AD > MCI > controls	The abundance of the *Enterobacteriaceae* family as a differential diagnostic tool for AD, MCI and healthy individuals.
Ling et al., 2021 [73]	Front. Cell Dev. Biol.	Humans: 100 AD patients and 71 age- and gender- matched healthy controls	16S rRNA Miseq sequencing of fecal microbiota	↓ Microbial diversity in AD compared to controls ↓ Butyrate producing bacteria (*Faecalibacterium*) ↑ Lactate producing bacteria (*Bifidobacterium*)	Microbiota shift from butyrate producer to lactate producer genera (from *Faecalibacterium* to *Bifidobacterium*)
Vogt et al., 2018 [114]	Alzheimer Res. Ther.	Humans: AD patients (*n* = 40), MCI patients (*n* = 35) and healthy controls (*n* = 335)	Cerebrospinal TMAO levels measurement	↑ TMAO in AD and MCI compared to controls	TMAO levels in the cerebrospinal fluid
Wu et al., 2021 [115]	Nutrients	Humans: AD patients (*n* = 27), MCI patients (*n* = 22) and healthy controls (*n* = 28)	LC/GC/MS metabolomics profiling of fecal microbiota	↓ Tryptophan metabolites in MCI and, more pronounced, in AD compared to controls ↓ SCFAs in MCI and, more pronounced, in AD compared to controls	Indole-3-pyruvic acid and five SCFAs for pre-onset and progression of AD

Abbreviations: APP/PS1: APPswe/PSEN1dE9 transgenic; GC: gas chromatography; LDA: linear discriminant analysis; LEfSe: linear disciminant analysis effect size; LC = liquid chromatography; MCI: mild cognitive impaired; MS: mass spectrometry; SCFAs: short chain fatty acids; 3xTg; triple-transgenic mouse model of AD; TMAO: Trimethylamine N-oxide; WT: wild type; ↑: increase; ↓: decrease.

**Table 2:** Evidence of diet as a possible complementary therapy in AD.

References	Type of Studies	Dietary Intervention	Aim	Outcomes
Duplantier et al., Nutrients, 2021 [166]	27 ObS, 5 RCT	Medi or DASH or MIND	Association between diet and cognitive health	Promising results for Medi diet but inconsistent outcomes. Lack of accuracy and standard tools
Bartochowski et al., Curr. Nutr. Rep., 2020 [167]	4 RCT	Medi or MIND	Association between diet and AD	Protective and promising therapeutic role of Medi. Not enough evidence for MIND.
	24 RCT	Vitamins and supplements (curcumin, EGb 761, EPA, DHA)		No statistically significant results; promising evidence for vitamin D supplementation and curcumin use.
Gutierrez et al., Nutrients, 2021 [168]	61 RCT	Different dietary patterns	Effects of nutrition on cognitive function	Healthy food consumption (Medi Diet) improves cognitive function. Polyphenols have protective effects. Low evidence for PUFAs, vitamin D and other supplements.
Limongi et al., J. Am. Med. Dir. Assoc., 2020 [169]	38 LS and 7 RCT	Medi	Association between diet and late-life cognitive disorders	Protective and promising therapeutic role of Medi diet for cognitive impairment.
Kheirouri et al., Critical Reviews in Food Science and Nutrition, 2021 [170]	9 CS, 3CrS, 1 RCT	MIND	Association between diet and neurodegenerative delay and cognitive functions	Improvement in cognition; limited number of studies and lack of mechanistic aspects in humans.
Lilamand et al., Curr. Opin. Clin. Nutr. Metab. Care, 2021 [171]	8 IS	KD or KS	Association between diet and cognitive and biological/neuropathological outcomes	Evident improvement: decrease in cerebral inflammation, Aβ-amyloid, aggregates of tau protein.
Grammatikopoulou et al., Adv. Nutr., 2020 [172]	10 RCT	KD or KS	Effects of KD on patients with AD/mild cognitive impairment	Improvement in acute and long-term cognition.
Pavón et al., Nutr. Rev., 2021 [173]	N/A	KD or KS	Effect of KD on cognitive skills in patients with AD, PD, refractory epilepsy, and type 1 glucose deficiency syndrome	Improvements in memory, cognitive performance and learning capabilities
Jensen et al., Int. J. Mol. Sci., 2020 [174]	N/A	KD or KS	Effects of KD on brain metabolism and function in neurodegenerative diseases	Reduction in AD symptoms.
Christensen et al., Nord. J. Psychiatry, 2021 [175]	24 RCT	KD or KS or modified Atkins diet	Effects of KD on CNS diseases	Modified-Atkins diet significantly improved memory in AD patients.
Moreira et al., Dement. neuropsychol., 2020 [176]	32 RCT	Omega-3, nutritional formula including ginseng, inositol and coconut oil	Association between diet and cognitive performance in AD	Omega-3 fatty acids showed positive effects at different doses. Probiotic, Ginseng, Inositol and specialized nutritional formulas might have a positive effect on cognition.
Zhang et al., Nutrients, 2020 [177]	12 CS, 3 case-control, 13 CrS, 1 IS	Meat	Association between meat (red meat, processed meat and poultry) consumption and cognitive functions	No significant association.
Dimache et al., Nutrients, 2021 [178]	21 (ObS, LS, CrS, IS)		Association between triglycerides with cognitive, vascular cognitive impairment and amyloid accumulation	In longitudinal studies: TG level is associated with cognitive decline. In cross sectional studies no correlation.
Gkotzamanis et al., Psychiatriki, 2020 [179]	4 RCT	Omega-3	Effect of supplementation on dementia	Promising preventative but not therapeutic effect.
	6 RCT	polyphenols		
El Gaamouch et al., Neurochem. Int., 2021 [180]	N/A	Grape polyphenols	Association between grape polyphenols and AD	No significant results from interventions.
Colizzi et al., Alzheimers Dement. (N Y), 2019 [181]	24 RCT	Polyphenols	Association between polyphenols and AD	12 studies found a positive correlation with reduced cognitive decline; 5 studies did not find any correlation and 7 studies reported mixed results.
Mielech et al., Nutrients, 2020 [182]	8 CS/RCT	Vitamins B	Association between antioxidant vitamins and AD and cognitive decline	4 studies: beneficial effect slowing cognitive decline; 4 studies: no differences
	3 CS/RCT	Vitamin A		Protective effect for cognitive functions in 2 studies.
	7 CS/RCT	Vitamins C and E		Protective effect for AD in 5 studies.
	7 CS/RCT	Vitamin D		Low level in the serum associated with increased risk of cognitive decline; no positive correlation with supplementation.
Szczechowiak et al., Pharmacology Biochemistry and Behavior, 2019 [183]	N/A	Pro-inflammatory (rich in saturated fats, meat) vs. anti-inflammatory (rich in vitamins, antioxidants, probiotics) diet	Association between pro- and anti-inflammatory diets and AD prevention and treatment	Overconsumption of foods rich in d-AGEs (Dietary Advanced Glycosylation End-products), saturated fats and red and processed meat have a pro-inflammatory influence on AD patients’ brains.
Kosti et al., Nutr. Rev., 2021 [184]		Fish, EPA/DHA supplementation	Associations between fish intake and AD dementia or AD and the effect of EPA/DHA supplementation on cognitive performance.	Regular consumption of fish up to 2 portions per week seems to be more protective than EPA/DHA supplementation.
Haider et al., International Journal of Geriatric Psychiatry, 2020 [185]	4 RCT	Vitamins B and E, omega-3, polyunsaturated fatty acids.	Effects of nutritional supplementation on neuropsychiatric symptoms among people with dementia	No significant results.
Arbo et al., Front. Aging Neurosci., 2020 [186]	3 RCT, 1 retrospective study	Resveratrol	Effect of resveratrol as potential treatment in AD and PD	No significant results in human trails.

Abbreviations: CrS: cross sectional study; CS: cohort studies; DHA: docosahexaenoic acid; EGb 761: Ginkgo biloba extract 761; EPA: eicosapentaenoic acid; KD: ketogenic diet; KS: ketogenic supplement; IS: interventional study; LS: longitudinal study; Obs: observational studies; RCT: randomized controlled trial.

**Table 3:** Murine and human studies performing FMT in AD.

Ref.	Journal	Study Cohort/Sample Size	Donor	Recipient	Transplantation Technique	Results
Hazan et al., 2020 [229]	J. Int. Med. Res.	Case study (*n* = 1)	85-year-old woman (recipient’s wife)	82-year-old man with recurrent CDI and AD	Single 300 mL FMT infusion	↑ Cognitive function (MMSE test) ↑ Memory ↑ Mood
Park et al., 2021 [230]	Curr. Med. Res. Opin.	Case study (*n* = 1)	27-year-old healthy man	90-year-old woman with AD and severe CDI	Colonoscopy (60 g of stool suspension for 2 times).	↑ Cognitive function tests (MMSE, MCA and CDR tests) ↑ Microbiota α diversity = Microbiota β diversity ↑ SCFAs
Kim et al., 2021 [231]	Brain. Behav. Immun.	Mouse (*n* = 8)	5xFAD mice	C57BL/6 mice	Oral gavage (200 ul for 5 consecutive days)	↓ Adult hippocampal neurogenesis and BDNF expression ↑ p21 expression ↑ Microglia activation ↑ TNF-α and IL-1β ↑Colon and plasma pro-inflammatory cytokines
Sun et al., 2019 [232]	Transl. Psychiatry	Mice (*n* = 8)	WT mice	APPswe/PS1dE9 transgenic (Tg) mouse model	Intragastrically (0.2 mL of fresh fecal solution once daily for 4 weeks)	↑ Cognitive function (MWM and ORT tests) ↓ Amyloid β brain deposition (Aβ40 and Aβ42) ↓ Tau protein phosphorylation ↑ Synaptic plasticity (increased PSD-95 and synapsin I) ↓ COX2 and CD11b ↑ SCFA and microbiota composition
Wang et al., 2021 [233]	Brain. Behav. Immun.	Mice (*n* = 4)	16 months old APP_SWE_/PS1_ΔE9_ mice	3 months old APP_SWE_/PS1_ΔE9_ mice	Antibiotic cocktails for 2 weeks by gavage and then FMT for 7 consecutive days by oral gavage	↑ Aβ plaques ↓ Astrocyte activation around Aβ plaques
Kim et al., 2020 [10]	Gut	Mice (*n* = 16)	WT mice	ADLP^APT^ transgenic mouse model	Fresh fecal matters for 16 weeks by oral gavage or for 4 weeks in mice pre-treated with antibiotics	↓ Aβ plaques ↓ Neurofibrillary tangles ↓ Glial reactivity ↓ Cognitive impairment ↓ Circulating blood inflammatory monocytes
Harach et al., 2017 [234]	Sci. Rep.	Mice (*n* = 6)	12 month-old) CONVR-WT or CONVR-APPPS1 mice	4 month-old GF-APPPS1 mice	Oral gavage of fecal contents on day 1 and day 4	↑ Cerebral Aβ pathology
Fujii et al., 2019 [235]	Biosc. Biotechnol. Biochem.	Humanized mice (*n* = 7)	4-weeks old germ-free C57BL/6N mice	Human healthy volunteers (76-year-old female) or AD patients (82-year-old male)	Oral inoculation	↓ OLT and ORT in mice colonized with AD microbiome ↓ γ-aminobutyrate, taurine and valine in mice colonized with AD microbiome
Zhan et al., 2018 [236]	Aging	Mice (*n* = 8)	SAMP8 or SAMR1 mice	pseudo germ-free mice	0.2 mL fecal suspension by gavage for 14 days	↑ Behaviour (only from SAMR1 transplant) ↑ α diversity and β diversity (only from SAMR1 transplant) ↓ Abnormal microbiota
Dodiya et al., 2019 [237]	J. Exp. Med.	Mice (*n* = 9)	age-matched APPPS1-21	ABX-treated APPPS1-21 male	0.2 mL fecal slurry by gastric gavage daily starting on P25 until sacrifice	↓ Aβ pathology ↑ Microglial physiology
Cui B. et al.,2018 [238]	Journal of Neuroinflammation	Mice (*n* = 6)	Low intensity noise (LN) exposure SAMP8 mice (control group) and high intensity noise (HN) exposure (AD model group)	male 3-month-old SAMP8 mice	0.1 mL fecal preparation via oral gavage twice per week for 30 days	↑ CLDN1 and ZO-1 in intestine and hippocampus of HN microbiota recipient ↑ Aβ in hippocampus of the HN microbiota recipient
Valeri et al., 2021 [239]	Microorganisms	Mice (*n* = 10)	Either 4 months old or 1 year old wild type mice	5xFAD mice (4-month old)	150 µ fecal preparation via oral gavage one time after antibiotics-treatment	↑ *Enterobacteriaceae*, *Lactobacillaceae*, serum LPS binding protein ↓ *Firmicutes* ↑ Plaques in dentate gyrus and prefrontal cortex

*ABX:* antibiotic cocktail; *APP_SWE_/PS1_L166P_*: APPPS1-21; BDNF: brain derived neurotrophic factor; CDI: *Clostridioides difficile* infection; CDR: Clinical Dementia Rating assessment; CLDN1: claudin 1; CONVR-APPPS1: conventionally-raised transgenic APPPS1 mice; COX2: cyclooxygenase 2; FMT: fecal microbiota transplantation; LPS: lipopolysaccharide; MCA: Montreal Cognitive Assessment; MMSE: Mini-Mental State Examination; MWM: Morris water maze test; OLT: object location test; ORT: object recognition test; PSD-95: postsynaptic density protein 95; SAMP8: senescence-accelerated mouse prone 8; SAMR1: senescence-accelerated mouse resistant 1; SCFAs: short chain fatty acids; ZO-1: Tight junction protein-1; ↑: increase; ↓: decrease.

## Data Availability

Not applicable.
